# Characteristics of TSPO expression in marmoset EAE

**DOI:** 10.1186/s12974-025-03343-4

**Published:** 2025-01-27

**Authors:** Irene Falk, Dragan Maric, Emily Leibovitch, Pascal Sati, Jennifer Lefeuvre, Nicholas J. Luciano, Joseph Guy, Seung-Kwon Ha, David R. Owen, Franklin Aigbirhio, Paul M. Matthews, Daniel S. Reich, Steven Jacobson

**Affiliations:** 1 Viral Immunology Section, National Institute of Neurological Diseases and Stroke, National Institutes of Health, Building 10, Room 5C103, 10 Center Drive, Bethesda, MD 20892-1400 USA; 2 Flow and Imaging Cytometry Core Facility, National Institute of Neurological Disorders and Stroke, National Institutes of Health, Bethesda, MD USA; 3 Translational Neuroradiology Section, National Institute of Neurological Disorders and Stroke, National Institutes of Health, Bethesda, MD USA; 4 Department of Neurobiology, University of Pittsburgh, Pittsburgh, PA USA; 5 Department of Brain Sciences, Imperial College London, London, UK; 6 Molecular Imaging Chemistry Laboratory, Wolfson Brain Imaging Centre, University of Cambridge, Cambridge, UK; 7 UK Dementia Research Institute, Imperial College London, London, UK

**Keywords:** TSPO, PET imaging, Multiple sclerosis, Experimental autoimmune encephalomyelitis

## Abstract

Multiple sclerosis (MS) is an inflammatory demyelinating disease of the central nervous system (CNS) and is a leading non-traumatic cause of disability in young adults. The 18 kDa Translocator Protein (TSPO) is a mitochondrial protein and positron emission tomography (PET)-imaging target that is highly expressed in MS brain lesions. It is used as an inflammatory biomarker and has been proposed as a therapeutic target. However, its specific pathological significance in humans is not well understood. Experimental autoimmune encephalomyelitis (EAE) in the common marmoset is a well-established primate model of MS. Studying TSPO expression in this model will enhance our understanding of its expression in MS. This study therefore characterizes patterns of TSPO expression in fixed CNS tissues from one non-EAE control marmoset and 8 EAE marmosets using multiplex immunofluorescence. In control CNS tissue, we find that TSPO is expressed in the leptomeninges, ependyma, and over two-thirds of Iba1 + microglia, but not astrocytes or neurons. In Iba1 + cells in both control and acute EAE tissue, we find that TSPO is co-expressed with markers of antigen presentation (CD74), early activation (MRP14), phagocytosis (CD163) and anti-inflammatory phenotype (Arg1); a high level of TSPO expression is not restricted to a particular microglial phenotype. While TSPO is expressed in over 88% of activated Iba1 + cells in acute lesions in marmoset EAE, it also is sometimes observed in subsets of astrocytes and neurons. Additionally, we find the percentage of Iba1 + cells expressing TSPO declines significantly in lesions > 5 months old and may be as low as 13% in chronic lesions. However, we also find increased astrocytic TSPO expression in chronic-appearing lesions with astrogliosis. Finally, we find expression of TSPO in a subset of neurons, most frequently GLS2 + glutamatergic neurons. The shift in TSPO expression from Iba + microglia/macrophages to astrocytes over time is similar to patterns suggested by earlier neuropathology studies in MS. Thus, marmoset EAE appears to be a clinically relevant model for the study of TSPO in immune dysregulation in human disease.

**Graphical Abstract:**

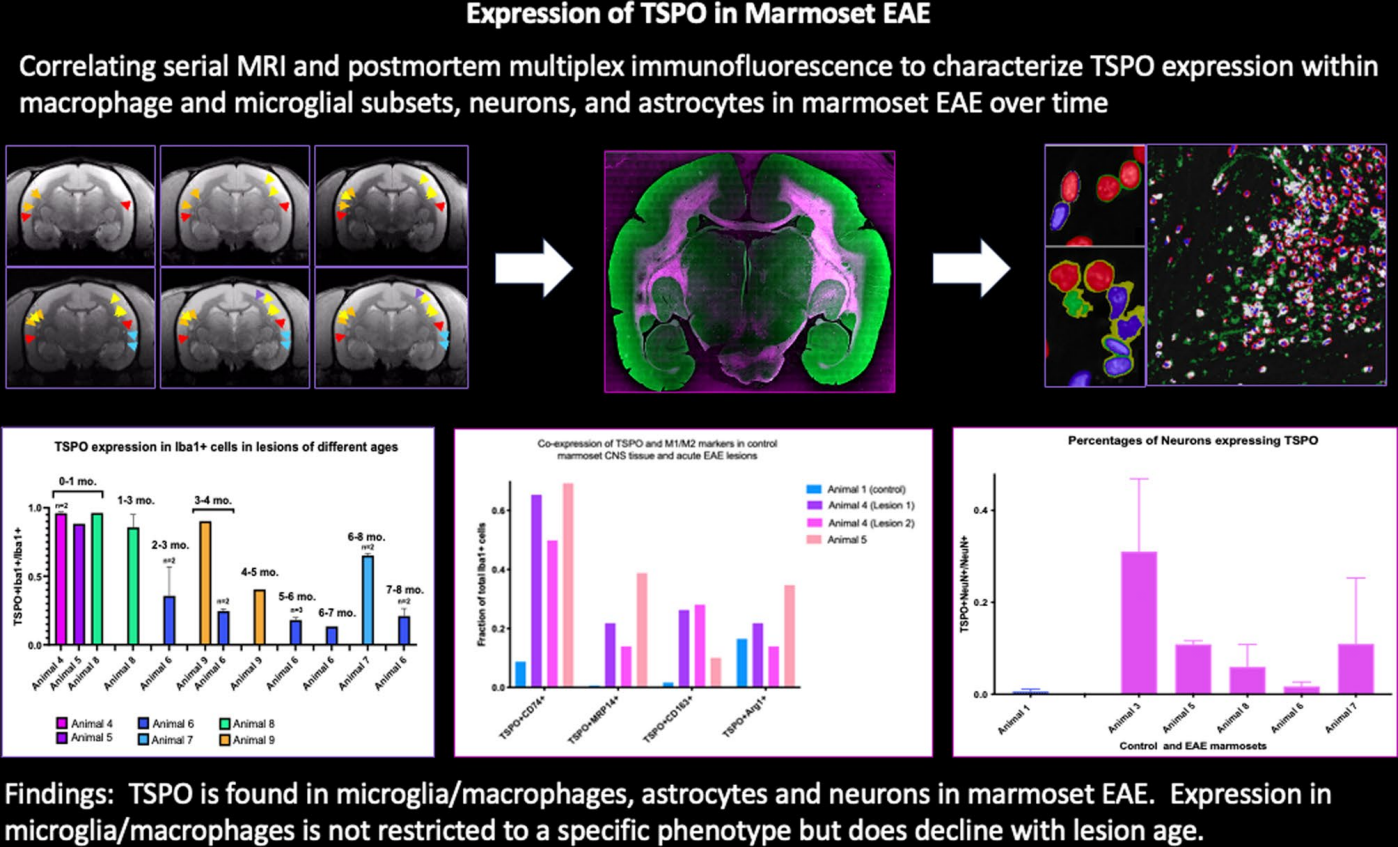

**Supplementary Information:**

The online version contains supplementary material available at 10.1186/s12974-025-03343-4.

## Introduction

Multiple sclerosis (MS) is a neuroinflammatory disease characterized by the infiltration of activated immune cells into the central nervous system (CNS), resulting in regions of focal axonal demyelination [1, 2]. Clinically, it is the leading cause of non-traumatic disability in young adults, affecting approximately 900,000 people in the U.S [3–5]. Radiologically, it is defined by CNS white matter lesions disseminated in time and space by magnetic resonance imaging (MRI), which is currently the gold standard of diagnosis [6]. However, MRI does not directly detect cellular mediators of inflammation.

One strategy for the direct detection of the chronic inflammatory activity associated with this disease is to use positron emission tomography (PET) radioligands targeting cellular markers of inflammation. The 18-kDa Translocator Protein (TSPO) is a mitochondrial transmembrane protein and widely used PET imaging target that has been studied extensively in a range of CNS pathologies, including MS [7–10]. Cells expressing TSPO densely accumulate in active and chronic CNS lesions in MS [11–13]. A 2017 study by Datta et al. [14] also found that MS patients with higher TSPO-PET signal in the normal-appearing white matter (NAWM) exhibited significant greater expansion of T2 hyperintense lesion volume on MRI at one-year follow-up, suggesting TSPO imaging may hold prognostic value for disease progression.

However, the pathophysiological significance of TSPO expression in this context is not well understood, and recent studies suggest significant differences in its expression and potential roles in human and rodent pathology. Whereas multiple rodent studies have found increased TSPO mRNA levels in lipopolysaccharide (LPS)-stimulated microglia [15–17], a 2017 study of human macrophages found *reduced* TSPO protein and mRNA levels after LPS-stimulation [18]. Similarly, while pathological studies of rodent EAE lesions have found increased density of TSPO staining in myeloid cells, no differences have been found in myeloid cells from MS lesions [19], and TSPO expression is observed in both pro- and anti-inflammatory microglia in MS brain tissue [13, 19–21].

Moreover, the different cell types contributing to TSPO expression in MS lesions have not been well defined. Studies in rodents and primates have shown increased TSPO expression in reactive astrocytes after CNS injury [9, 22–26], but the presence and extent of this upregulation appear to vary significantly between models [9, 10]. Murine studies demonstrate microglia to be the principal source of TSPO density in neuroinflammatory disease [15], and certain rat models of EAE appear to lack astrocytic TSPO expression altogether [23, 25]. Studies in MS, in contrast, have found astrocytes to be major contributors of TSPO, particularly in chronic and chronic active lesions [13], and it is hypothesized that astrocytic TSPO may serve a neuroprotective role by providing increased neurotrophic support [9, 26].

TSPO expression has also been identified in neurons cultured in vitro, including human neuroblastoma and glioblastoma cell lines, primary cortical neurons and cerebellar granule cells in mammals, and dorsal root ganglia sensory neurons in rats [27–29]. A 2009 study of a murine neural stem cell line detected TSPO protein expression and increased mRNA levels in differentiating and regenerating neuronal precursor cells at an intermediate stage of maturation [27], suggesting TSPO may play a role in neuronal maturation.

EAE is an animal model of MS that is commonly induced by immunizing experimental animals with myelin-derived proteins such as myelin-oligodendrocyte glycoprotein (MOG) or proteolipid protein (PLP) [30]. While EAE is most commonly studied in mice, a significant challenge of this approach is the lack of murine models to recapitulate the spectrum of radiological and pathological manifestations seen clinically in MS [31]. Serial MRI analysis is valuable imaging modality for the non-invasive assessment of lesion load, disease progression, and therapeutic response over time, but mice possess less white matter than primates, which poses a limitation to the use of MRI monitoring in murine EAE progression [32]. Consequently, increasing attention is being paid to primate models of EAE, and the common marmoset has emerged as a useful model for addressing these limitations.

As primates, marmosets possess a neural architecture that is functionally and structurally like that of humans [32, 33]. Radiologically, EAE marmosets can manifest perivenular white matter lesions identifiable by MRI [34, 35], another feature of human disease typically absent in murine models [32]. They are also more immunologically similar [31, 32, 34, 36, 37]. Bred in colonies, marmosets acquire a diverse repertoire of immune exposures (in contrast to pathogen-free rodents [33]), and marmoset EAE lesions exhibit features of human disease not as frequently seen in murine EAE lesions, such as B cell infiltration and CD8 + T cell involvement [32, 34]. Clinically, marmoset EAE typically manifests with an early relapsing-remitting course followed by a chronic progressive phase [31]. Studying TSPO in marmoset EAE would facilitate the serial comparison of PET-TSPO and white matter lesion load on MRI, enabling the analysis of disease progression from both radiological and clinical standpoints. Data we have previously published with Nutma et al. demonstrate that like myeloid cells in MS lesions, myeloid cells in marmoset EAE do not exhibit increased TSPO density when compared to normal appearing tissue, but total TSPO density increases, largely due to increased TSPO + microglia/macrophage cell density [19]. However, we have not previously had the opportunity to compare TSPO expression in marmoset EAE directly with non-EAE control tissue, and the range of cell phenotypes and lineages expressing TSPO in marmoset EAE has not yet been characterized.

In this paper, we characterize and compare the nature of cells expressing TSPO in the CNS tissues of non-EAE and EAE-affected marmosets, which we find more comparable to patterns of distribution seen in humans than those seen in rodents. In control tissue, TSPO expression is seen in the leptomeninges, ependyma, and three-quarters of all Iba1 + microglia, but not neurons or astrocytes. In EAE lesions < 4 weeks old, we find that the percentage of Iba1 + microglia/macrophages expressing TSPO within lesions is increased. While activated microglia/macrophages contribute the majority of TSPO in these early EAE lesions, this contribution declines over time. Moreover, TSPO is also observed in astrocytes in lesions with astrogliosis and some neurons in the cortical gray matter. We find that TSPO is not restricted to a single microglial phenotype, regardless of disease status. This pattern of TSPO expression in marmoset EAE closely resembles that described earlier for MS, suggesting that it provides a clinically relevant model for the study of TSPO and TSPO-PET imaging.

## Materials and methods

### EAE induction and animal monitoring

EAE was induced by subcutaneous immunization with 0.2 g of white matter homogenate emulsified in CFA in 9 adult marmosets at 4 dorsal sites adjacent to inguinal and axillary lymph nodes. Animals were monitored daily for clinical symptoms of EAE progression and assigned clinical EAE scores weekly based on extent of disability. Neurological exams were performed by a neurologist prior to each MRI scan. The scoring system is as follows: 0, no clinical signs; 0.5, apathy or altered ambulation without ataxia; 1, lethargy or tremor; 2, ataxia or optic disease; 2.25, monoparesis; 2.5, paraparesis or sensory loss; 3, paraplegia or hemiplegia. Body weights were recorded 3 times per week. A clinical score of 3 was predefined as a study endpoint. Animals #8 and #9 were treated with prednisolone for 5 days as part of a concurrent study [38]. These animals were the first within their twin pair that showed three or more brain lesions by in vivo MRI and received corticosteroid treatment with the goal to reduce the severity of inflammation and potentially allow longer-term evaluation of the lesions. Animal #9 received 7.5 mg of prednisolone 189 days prior to sacrifice (perfusion). All activities were performed in accordance with NIH-IACUC protocols. All animals discussed in this study are shown in Table 1.

**Table 1:** Table summarizing gender, disease status, age at baseline, and disease duration for all animals examined in this study

Animal ID	Gender	Disease Status	Age at EAE induction (years)	Disease duration (days)
1	M	Control	N/A	N/A
2	F	EAE	1.5	89
3	M	EAE	3.9	71
4	F	EAE	4.6	32
5	F	EAE	2.9	105
6	M	EAE	2.6	384
7	M	EAE	5.6	422
8	F	EAE	2.9	123
9	M	EAE	2.6	282

### MRI scanning

Animals were anesthetized and imaged according to previously published marmoset imaging protocols using T1, T2, T2*, and PD-weighted sequences on a Bruker 7T animal magnet [33, 37, 39, 40]. Marmosets were scanned biweekly over the course of the EAE study. Following EAE study completion, the brains, spinal cords, and optic nerves excised from euthanized animals were scanned by MRI for post-mortem characterization of brain lesions and previously uncharacterized spinal lesions and optic nerve lesions. Lesions < 1 month old at time of sacrifice were defined as acute, while lesions 1–3 months old were defined as subacute and lesions > 3 months old as chronic.

### Immunohistochemistry

To characterize TSPO expression in microglia/macrophages in EAE lesions, marmoset CNS tissues from healthy or EAE-affected animals were formalin-fixed and paraffin-embedded. For immunohistochemistry, paraffin-embedded sections were deparaffinized in three changes of xylene, rehydrated in three changes of graded ethanol, and washed in ultrapure water.

Antigen retrieval was performed by steaming the slide in 10 mM citrate buffer (pH 6) for 20 min. Sections were washed with tris-buffered saline (TBS), blocked for 20 min in 10% non-fat dairy protein in TBS, and again washed with TBS. For diaminobenzidine (DAB) staining, sections were incubated with a horseradish peroxidase-conjugated secondary antibody diluted 1:2000 in TBS for 15 min at room temperature and then incubated with DAB chromogen substrate from a DAB substrate kit according to the manufacturer’s instructions (Vector Laboratories). DAB-stained sections were differentiated in Blue Buffer and counterstained with hematoxylin (Leica Biosystems). For Luxol Fast Blue (LFB) staining, sections were incubated overnight in NovaUltra LFB Solution at 56 C, differentiated with lithium carbonate, and counterstained with periodic acid-Schiff (PAS) (IHC World). Following substrate visualization, sections were washed in TBS and coated with Immu-Mount (Fisher, 99-904-02), after which each section was sealed with a 22 × 50 mm coverslip.

### Multiplex immunofluorescence

To immunophenotype microglia/macrophages expressing TSPO in the CNS, a 7-color multiplex immunofluorescence panel was used to stain for Iba1, PLP, TSPO, MRP14, CD74, CD163, and Arg1.

Deparaffinized sections were washed twice in PBS supplemented with 1 mg/ml BSA (PBS/BSA), followed by two washes in distilled water. Antigen retrieval was performed by boiling the slide in 10 mM citrate buffer (pH 6) for 10 min in an 800 W microwave at maximum power, after which they were allowed to cool for 30 min and washed twice in distilled water. To reduce nonspecific Fc receptor binding, the section was incubated in 250 µl of FcR blocker (Innovex Biosciences, cat. no. NB309) for 15 min at room temperature and washed twice in distilled water. To further reduce background, sections were coated with 250 µl Background Buster (Innovex Biosciences, cat. no. NB306) for 15 min at room temperature and washed twice in distilled water. Sections were incubated for 45 min at room temperature in a primary antibody cocktail containing antibodies diluted in PBS/BSA (Supplemental Table 1), washed in PBS/BSA and three changes of distilled water. They were then incubated for 45 min in a secondary antibody cocktail composed of secondary antibodies diluted in PBS/BSA containing DAPI (Invitrogen, cat. no. D1306, 100 ng/ml) (Supplemental Table 2), then washed once in PBS/BSA and twice in distilled water. To facilitate mounting, the sections were air-dried for 15 min at room temperature, sealed with a coverslip as described previously, and allowed to dry overnight prior to image acquisition.

### Antibody stripping of previously stained sections

To study the expression of TSPO in reactive astrocytes and neurons, selected sections were stripped and re-stained for Iba1, GFAP, calcium binding astrocytic marker S-100-B, neuronal nuclear marker NeuN, neuronal dendritic marker MAP2a, and axonal neurofilament NFH. To facilitate re-staining, coverslips were removed carefully from mounted sections, and the uncovered sections were incubated for 10 min at room temperature in 250 µl of NewBlot Nitro 5X Stripping Buffer (Li-Cor, cat. no. 928-40030) to remove tissue-bound antibodies from the first round of immunostaining. The stripped sections were washed once in PBS/BSA, followed by two washes in distilled water.

To detect antigens not restored by prior antigen retrieval, sections were boiled for an additional two minutes in citrate buffer (pH 6.0) in an 800 W microwave at 100% power, after which the sections were allowed to cool for 30 min and washed twice in distilled water. FcR blocking and nonspecific blocking were performed as described previously. Sections were incubated in a cocktail of primary antibodies (Supplemental Table 3) and washed as described previously. They were then incubated in the appropriate secondary antibody cocktail (Supplemental Table 4), followed by nuclear counterstaining with DAPI and cover-slipping, as described previously.

### Image acquisition

Sections were imaged using a Zeiss AxioImager.Z2 widefield scanning microscope with a 20X/0.8 Plan-Apochromat (Phase-2) non-immersion objective (Zeiss), a high resolution ORCA-Flash4.0 sCMOS digital camera (Hamamatsu), a 200 W X-Cite 200DC broad-band lamp source (Excelitas Technologies), and 7 customized filter sets (Semrock) optimized to detect the following fluorophores: DAPI, AlexaFluor 430, AlexaFluor 488, AlexaFluor 546, AlexaFluor 594, AlexaFluor 647 and IRDye 800CW. 16-bit monochrome image tiles (600 × 600 μm area) were individually captured at a 0.325 μm/pixel spatial resolution and stitched into whole-specimen images using the ZEN 2 image acquisition and analysis software program (Zeiss).

### Image quantification

To quantify TSPO in Iba1 + cells, an image segmentation algorithm was used to identify Iba1 + cells with visible DAPI + nuclei, thereby restricting analysis to Iba1 + microglia/macrophages, while excluding Iba1 + cell fragments, apoptotic debris, and DAPI + nuclei not associated with Iba1. To count neurons, 500 μm x 500 μm regions of interest (ROI) were selected in the right and left cortical gray matter, and cells expressing NeuN, TSPO, glutaminase 2 (GLS2), or parvalbumin (PVA) alone or in combination were counted manually.

### Statistical analyses

To study changes in TSPO expression over time in lesions of different ages, of which a subset of lesions were found in the same animal, we performed a changepoint analysis using a linear mixed-effects model, with time point as a categorical independent variable, to account for the non-independence of these data points [41, 42]. The fraction of Iba1 + cells expressing TSPO in each lesion was modeled as a function of lesion age at time of sacrifice. Observations comprise lesions within animals and are further designated by a binary indicator distinguishing prednisolone-treated animals from untreated animals. Random effects comprise random variation between animals and random variation between lesions within single animals. Fixed effects consist of months-prior-to-sacrifice and the binary indicator for prednisolone treatment. To determine the potential role of steroid treatment in this model, an alternative parameterization was used to estimate the fraction of Iba1 + cells that are TSPO + in the absence of steroid treatment over all months before sacrifice by calculating successive differences from the cumulative average of the preceding values up to that time, e.g., subtracting the TSPO+/Iba1 + ratio three months prior to sacrifice from the average of the TSPO+/Iba1 + ratios at one and two months prior to sacrifice. Animal identity, treatment status, and lesion age for each lesion are summarized in Supplemental Table 5.

The fraction of neurons expressing TSPO in control animal #1 and 5 EAE animals was compared by assessing ROIs from the left and right cortex. The effect of disease status was assessed against between-animal variation using a linear mixed effects model with a random effect for animal identity and fixed effects for EAE status and the laterality of the ROI. Each measurement was weighted by the number of cells per ROI.

Analysis was performed in R (R Foundation for Statistical Computing, Vienna, Austria). A p-value < 0.05 was considered statistically significant. The R code used to perform this analysis was derived from analyses by Kusnetsova et al. [42, 43].

## Results

### TSPO is expressed in the leptomeninges, ependyma, vascular endothelium, and a subset of microglia in control CNS tissue

**Fig. 1:**
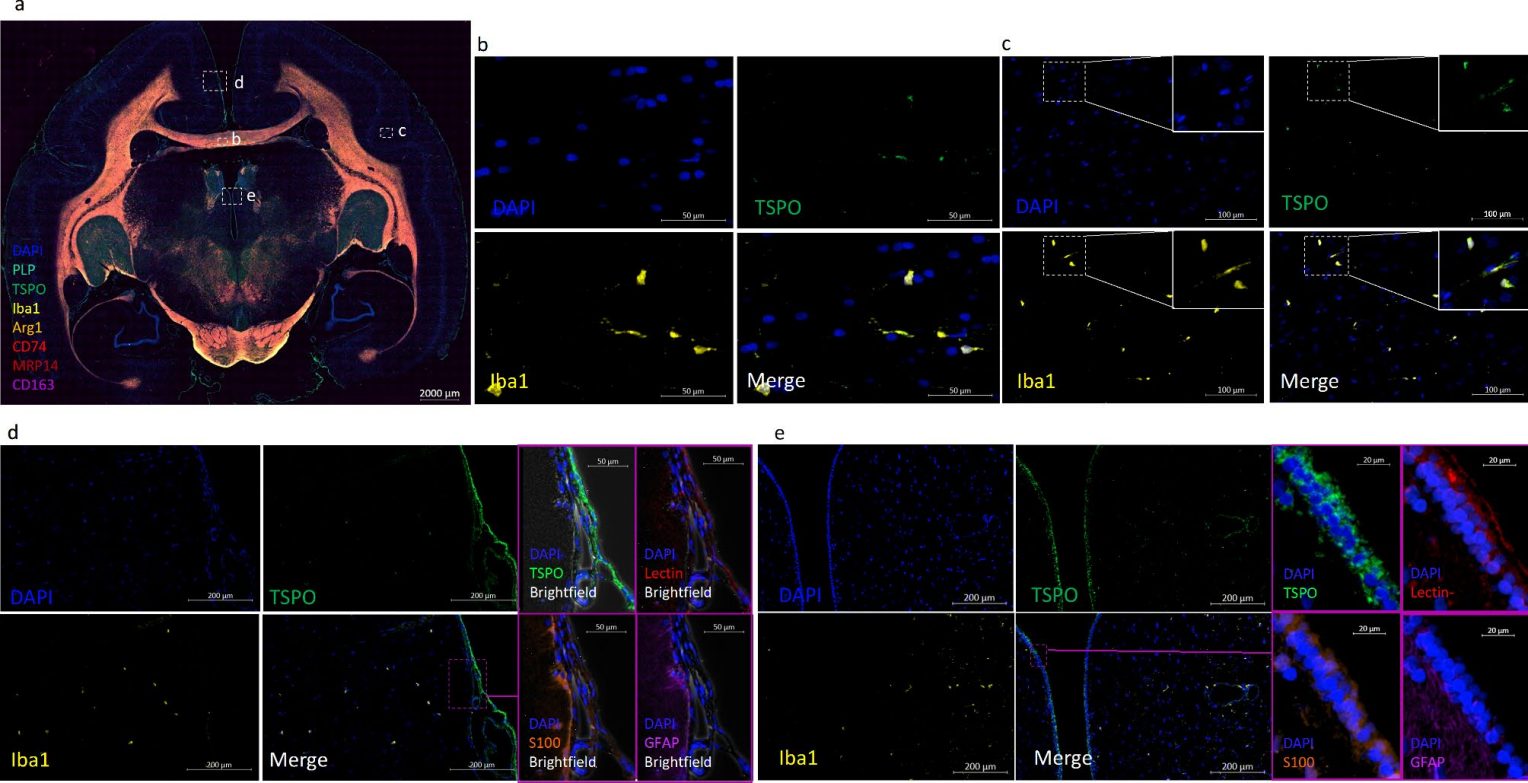
In non-EAE control CNS tissue, TSPO is expressed in the leptomeninges, ependymal cells, subarachnoid vascular endothelium, and a subset of Iba1 + microglia, but not the astrocytes of the glia limitans. 1**a**) Multiplex staining of a section of brain tissue from non-EAE control animal #1, with regions of interest b-e indicated by white outlines. 1**b**-**c**) TSPO, Iba1, and DAPI staining in the b) NAWM and c) NAGM of non-EAE control animal #1, showing TSPO expression in a subset of Iba1 + cells. 1**d**-**e**) TSPO, Iba1, and DAPI staining showing TSPO expression in the d) meninges and e) ependyma of non-EAE control animal #1, with insets showing TSPO vs. lectin, S100, and GFAP staining after antibody stripping and re-straining. TSPO is observed to co-localize with lectin in the leptomeninges external to the subarachnoid space and vascular endothelium in the subarachnoid space but not the GFAP + and S100 + cells of the glia limitans. It is also observed to co-localize with S100 in the simple columnar epithelium on the ependyma

To determine the identity of TSPO-expressing cells in marmoset CNS tissue, we applied the IHC-validated multiplex immunofluorescence panel described in Supplemental Tables 1–4 to sections of brain and spinal cord tissue from EAE marmosets and one control marmoset (Table 1). Different cell types expressing TSPO in control CNS tissue are shown in Fig. 1. Figure 1a shows a section of control brain tissue from animal #1 stained with TSPO, Iba1, CD74, MRP14, CD163, Arg1, PLP, and DAPI, with ROI labeled. Figure 1b-e show TSPO, Iba1, and DAPI staining within each ROI. TSPO expression can be seen in Iba1 + cells in the white matter (WM) of the corpus callosum (Fig. 1b) and gray matter (GM) of the left cortex (Fig. 1c). Several of these Iba1 + TSPO + cells are shown at higher resolution (Fig. 1c, **inset**, arrows). TSPO staining can also be seen clearly in Iba1- cells in the leptomeninges and subarachnoid vessels lining the interhemispheric fissure (Fig. 1d). Comparison of TSPO staining with GFAP, S100, and lectin staining after re-staining of the same section demonstrates that TSPO co-localizes with lectin + cells in the leptomeninges, particularly the arachnoid mater external to the subarachnoid vessels, and the endothelial cells of the vessels themselves. Figure 1e demonstrates TSPO staining in ependyma of the third ventricle and the vascular endothelium of a blood vessel in the adjacent gray matter. Specifically, TSPO is observed to co-localize with S100 in the simple cuboidal and columnar epithelium of the ependymal cells lining the ventricle (Fig. 1e, inset). However, TSPO does not appear to be expressed in astrocytes in control tissue. No TSPO is seen in the S100 + or GFAP + cells of the glial limitans [44] (Fig. 1d, inset) or in the gray matter adjacent to the ventricle (Fig. 1e, inset).

### TSPO density is increased in marmoset EAE lesions relative to control CNS

**Fig. 2:**
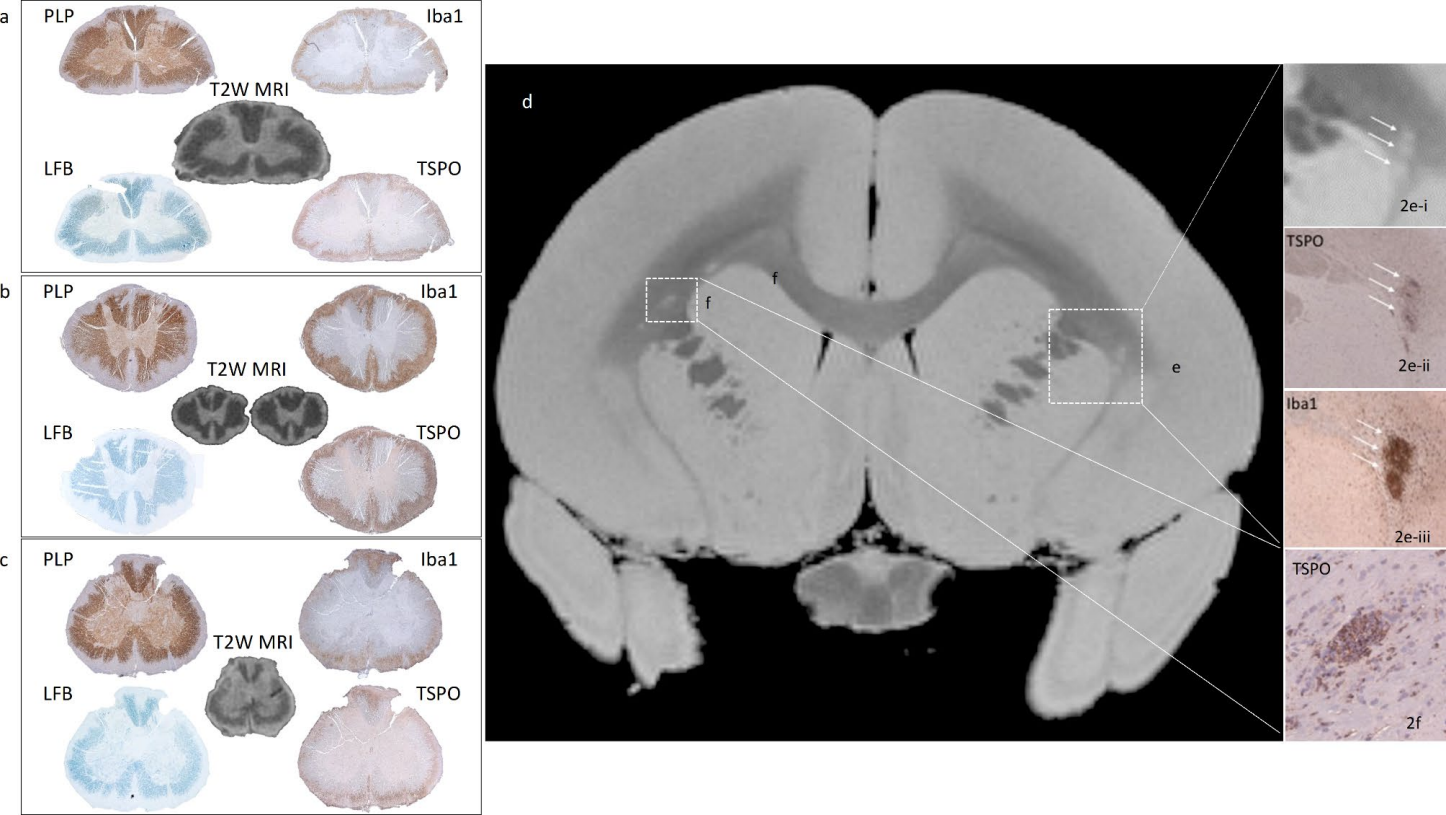
TSPO density corresponds with Iba1 + density in demyelinated lesions established by T2*-weighted gradient echo MRI, LFB staining, and PLP staining in EAE brain and spinal cord tissue. 2**a**-2**c**) Comparison of PLP, LFB, Iba1, and TSPO staining with T2*-weighted gradient echo MRI in the (**a**) cervical, (**b**) thoracic, and (**c**) lumbar spinal cord of EAE marmoset #2. Iba1 and TSPO staining was visualized with DAB chromogen and hematoxylin counterstaining. TSPO density is observed to correlate with demyelination seen on LFB and PLP staining as well as the accumulation of microglia/macrophages indicated by Iba1 staining. 2**d**) Post-mortem proton density (PD)-weighted MRI from EAE marmoset #3, with three adjacent left white matter lesions (inset e, white arrows) and one right white matter lesion (inset f, white arrows). 2**e**) Post-mortem PD-weighted MRI (i), TSPO (ii), and Iba1 (iii) within the lesions in area e. 2**f**) TSPO staining in lesion area f, which is also observed to correlate with demyelination on PDW MRI and Iba1 staining shown in 2e. PLP, Iba1 and TSPO staining was visualized with DAB chromogen and hematoxylin counterstaining

Fixed sections of brain and spinal cord tissue from EAE marmosets were stained by conventional immunohistochemistry for TSPO. Staining from the spinal cord of animal #2 is shown in Fig. 2. LFB staining was performed on contiguous sections to visualize an extensive circumferential subpial lesion seen in the white matter at all levels of the spinal cord. Contiguous serial sections were also stained for Iba1 to visualize macrophages and microglia and PLP to visualize demyelination in both gray and white matter. In acute (< 1 month old), subacute (1–3 months old) and chronic (> 3 months old) EAE lesions, there was an increase in the density of Iba1 + microglia/macrophages and the density of TSPO relative to control tissue (Fig. 1**)**. PLP and LFB staining revealed clusters of demyelinated lesions in all EAE animals, while Iba-1 staining showed dense macrophage/microglia accumulation within these lesions, which were also highly positive for TSPO. A comparison of Iba1, PLP, LFB, and TSPO staining in the spinal cord of EAE marmoset #2 is shown in Fig. 2a-c, in which TSPO staining was found to correspond spatially with Iba1 staining and demyelination detected by PLP and LFB staining in the cervical (a), thoracic (b), and lumbar (c) spinal cord. These lesions were also found to correlate to hyperintensities identified on post-mortem T2*-weighted gradient-echo MRI.

**Fig. 3:**
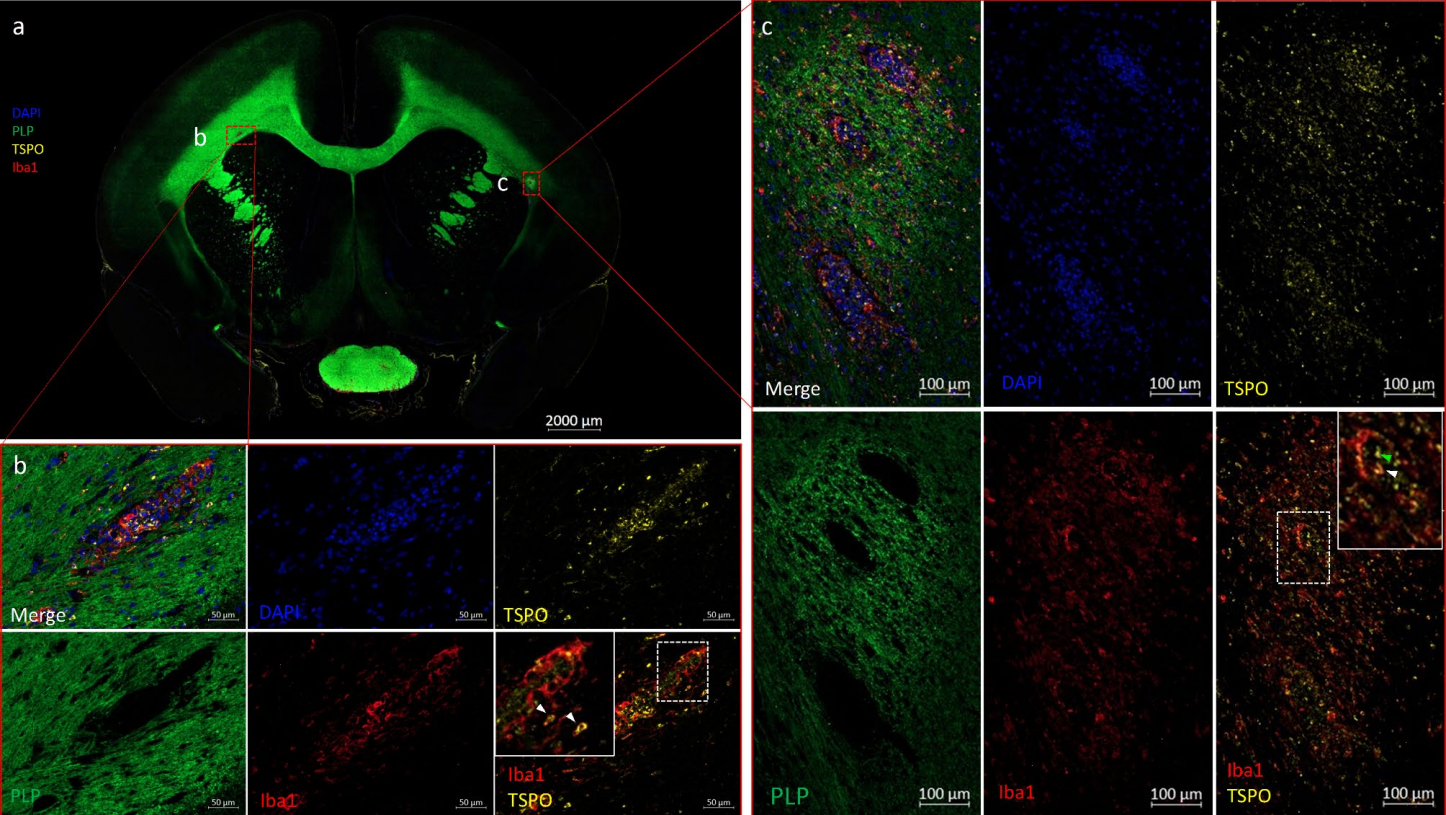
Multiplex immunostaining confirms that TSPO is expressed in Iba1 + cells in marmoset EAE but is also expressed by Iba1- cells. 3**a**) Post-mortem multiple staining from EAE marmoset #3, with three adjacent left white matter lesions (area b, white arrows) and one right white matter lesion (area c, white arrows). 3**b**) PLP, Iba1, DAPI, and TSPO staining in area b, with inset showing two TSPO + Iba1 + cells (white arrowheads). 3**c**) PLP, Iba1, DAPI, and TSPO staining in area c, with inset showing a TSPO + Iba1 + cell (white arrowhead) and an TSPO + Iba1- cell (green arrowhead)

Lesions identified on proton density (PD)-weighted MRI in the white matter of EAE marmoset #3 (Fig. 2d, white boxes) were also found to correlate with increased TSPO and Iba1 staining, as shown in Fig. 2e-i **-iii**, in which three lesions are indicated by white arrows on: (i) proton density-weighted MRI, (ii) TSPO staining, and (iii) Iba1 staining. As in the control tissue, multiplex fluorescent staining demonstrated co-localization of TSPO in Iba1 + cells in these lesions; multiplex fluorescent staining is shown in Fig. 3a, and TSPO, Iba1, PLP, and DAPI staining in selected areas are shown in Fig. 3b (demyelinated lesion) and Fig. 3c (active lesion containing areas of demyelination surrounded by myelin debris).

**Fig. 4:**
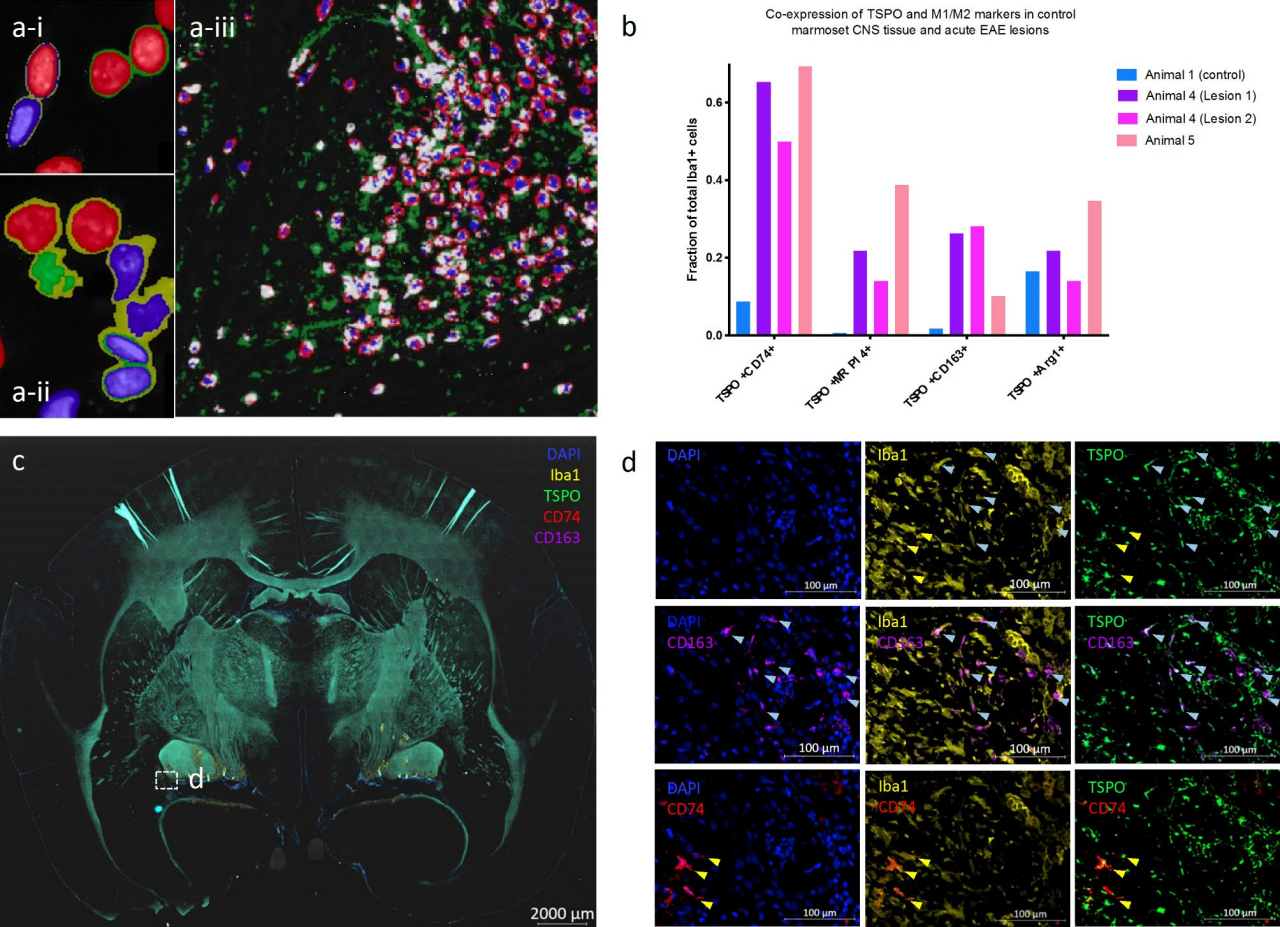
TSPO colocalizes with markers associated with early activation, antigen presentation, phagocytosis, and immunosuppression in marmoset EAE and is not restricted to a particular phenotype. 4a.i-4a.ii) Representative segmentation of DAPI + nuclei into irregular cell clusters (yellow outline), which are further segmented at higher thresholds to identify spherical nuclei (red), elliptical nuclei (blue), and bilobed shapes composed of two overlapping nuclei (green outline). Bilobed clusters are further segmented to identify the individual nuclei within the cluster. **a-iii)** Cell masks for microglia and macrophages are created by scanning for contiguous Iba1 + pixels (red) within a 15-pixel radius from the edge of the DAPI + nucleus (blue). The cell mask resulting from the addition of DAPI + and Iba1 + pixels are then scanned for TSPO (white). TSPO pixels following outside the DAPI + Iba1 + cell mask, i.e., TSPO expressed by other cell types, are shown in green. The same DAPI + Iba1 + mask is then scanned for other markers (CD74, CD163, Arg1, MRP14) to determine which are co-expressed with TSPO. **b)** Comparison of fractions of Iba1 + cells co-expressing TSPO and other markers of interest (CD74, CD163, Arg1, MRP14) in a control marmoset and 3 acute lesions from EAE marmosets #4 and #5. Two acute lesions were identified in animal #4, while one lesion was identified in animal #5. **c)** DAPI, Iba1, CD163, CD74, and TSPO staining in EAE animal #4, with region d indicated below the left optic tract. **d)** DAPI, Iba1, CD163, CD74, and TSPO staining in region d, showing that TSPO co-localized with both CD163 (blue arrows) and CD74 (yellow arrows)

To better quantify the density and phenotypic associations of TSPO in marmoset EAE, we examined the expression of TSPO and markers of immunophenotype in control CNS tissue from animal #1 and acute EAE lesions (*n* = 3) from EAE animals #4 and #5. Segmentation of Iba1 + cells with visible DAPI-stained nuclei identified 34,345 microglia/macrophages within a 19.8 mm x 16.2 mm section of control primate brain and approximately 8,000–10,000 microglia/macrophages in each of the acute lesions. Figure 4a-i and a **-ii** show representative segmentation of DAPI + nuclei into spherical nuclei (red), elliptical nuclei (purple), and bilobed shapes composed of two overlapping nuclei (green outline) or irregular cell clusters (yellow outline), which are further segmented into individual cells. Figure 4a **-iii** shows the definition of a microglia/macrophage cell mask composed of DAPI + nuclei (blue) and contiguous Iba1 + pixels (red), which are then scanned for intracellular TSPO (white). TSPO + pixels not associated with Iba1 + nuclei appear green. The density of Iba1 + cells in the control animal as 1.13 cells per 100,000 pixels. Quantification of TSPO + pixels with an intensity above a selected threshold (set by manual inspection to exclude acellular or nonspecific staining) indicated TSPO expression above threshold in 76.5% of Iba1 + cells in the control CNS, or 0.86 cells per 100,000 pixels.

Each Iba1 + cell body was also then scanned for expression of early activation marker MRP14 [48], MHC-II molecule HLA-DR [20, 46], phagocytic receptor CD163 [50, 51], and Arg1, which is associated with immunosuppression [49]. We found that TSPO was expressed in association all four markers, indicating that it is expressed in cells with both immunogenic and/or tolerogenic functions; it is not restricted to microglia of a particular phenotype. The fractions of total Iba1 + microglia/macrophages expressing TSPO and another marker of immunophenotype (i.e., TSPO + CD74+, TSPO + Arg1+, TSPO + CD163+, or TSPO + MRP14 + cells) in control animal #1 and acute-to-subacute lesions from animals #4 and #5 are shown in Fig. 4b. Figure 4c shows DAPI, Iba1, TSPO, CD74, and CD163 staining from animal #4 with an ROI defined caudal to the left optic tract. This region is shown at higher resolution in Fig. 4d, where colocalization of TSPO with both CD74 (blue arrows) and CD163 (yellow arrows) is apparent. We found that TSPO is more frequently expressed by Iba1 + cells expressing at least one additional marker of activation (e.g. Arg1, CD74, CD163, or MRP14), and that the frequency of this co-expression increases in EAE. In control animal #1, TSPO was detected in 85.1% of Iba1 + cells expressing at least one of the four phenotypic markers used in this panel but only 30.3% of cells that did not express any additional activation marker (i.e. Arg1-CD163-CD74-MRP14- cells). Of the presumed microglia that expressed TSPO, 22.1% expressed Arg1, but only 11.2% expressed antigen presentation marker CD74 and less than 0.05% expressed early activation marker MRP14.

In EAE tissue, the densities of Iba1 + cells within lesions < 4 weeks old ranged from 6.8 to 9.9 cells per 100,000 cells as previously reported [19], roughly 5- to 10-fold higher than observed in control tissue. Within these lesions, upwards of 88% of Iba1 + cells were also TSPO+, and substantial increases were observed in the expression of CD74, MRP14, and CD163, but not Arg1. For example, HLA chaperone protein CD74 was co-expressed with TSPO in only 8.1% of Iba1 + microglia in the control marmoset tissue but over 50% of Iba1 + cells in lesions < 4 weeks old (Fig. 4b). There were also marked increases in both the absolute number of CD163 + microglia/macrophages and the fraction of microglia/macrophages co-expressing TSPO and CD163 (1.7% of Iba1 + cells in the control brain vs. over 20% of Iba1 + cells in lesions < 4 weeks old). MRP14, which was expressed with TSPO in less than 0.05% of Iba1 + cells in control brain tissue, was expressed with TSPO in at least 14% of Iba1 + cells in lesions < 4 weeks old (Fig. 4b). While Arg1 was the most commonly detected immunophenotypic marker in control brain tissue, its fractional expression did not appear to increase to the same degree as that of the CD74, MRP14, or CD163. In these lesions, Arg1 was co-expressed with TSPO in, on average, 22.5% of Iba1 + cells, vs. 16.5% of Iba1 + cells in control animal #1 (Fig. 4b). The number of cells expressing each distinct combination of markers and the percentage of those cells expressing TSPO in animals #1, #4 and #5 are shown in Supplemental Tables 6–8.

### The fraction of Iba1 + macrophages/microglia expressing TSPO declines with lesion age

**Fig. 5:**
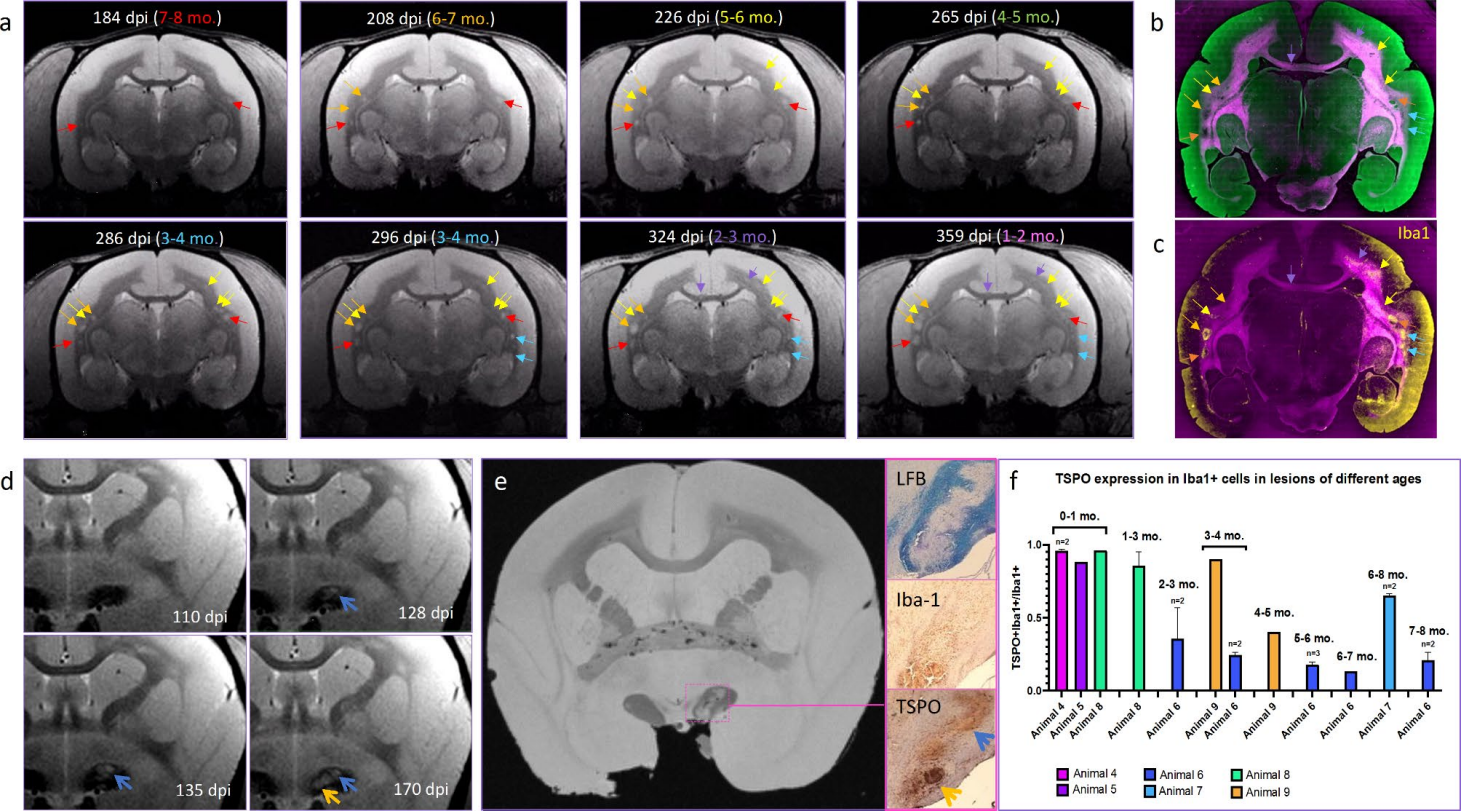
The fraction of Iba + microglia/macrophages expressing TSPO declines with lesion age. 5 **a**) Serial proton density-weighted MRIs from EAE marmoset #6 at various time points (days post-induction, dpi) over an 8-month period prior to sacrifice, on which lesions are identified according to age at sacrifice by color as follows: 2–3 mo. — purple; 3–4 mo.— blue, 4–5 mo. – green, 5–6 mo. — yellow, 6–7 mo. — orange, 7–8 mo. — red. **5b)** Arrows indicating lesions identified from the MRI slices in 5a are overlaid on a corresponding tissue section, from which autofluorescence from channel 9 (pink) and background signal from channel 4 (green) are shown for maximal contrast to visualize structural changes in the white matter. **5c)** Arrows indicating lesions identified from the MRI slices in 5a are overlaid on Iba1 and PLP staining from the corresponding tissue section to visualize microglia/macrophages and demyelination. **5d)** Post-mortem T2*-weighted imaging of EAE marmoset 9, with LFB, PLP, and TSPO staining of two lesions in the left optic tract shown in the inset, one first identified at 128 dpi (blue arrow), and a second identified at 170 dpi (orange arrow). TSPO and Iba1 staining are markedly more intense in the younger lesion identified at 170 dpi. **5e)** Fractions of Iba1 + cells co-expressing TSPO in lesions of different ages from 6 EAE marmosets, showing a decline in the expression of TSPO in chronic lesions > 4 months old at time of sacrifice. Error bars indicate SEM

To further probe the time course of marmoset EAE, a multiplex immunofluorescence protocol was applied to sections of brain tissue from six EAE animals with lesions of known age, ranging from < 4 weeks old to 7–8 months old. Figure 5a shows an example of serial MRI with representative lesion staging from EAE animal #6. The MRI demonstrates multiple lesions ranging from 2 to 3 months to 7–8 months in age at time of sacrifice (Fig. 5a, arrows). Figure 5b-c show the same lesions identified on a tissue section corresponding to this MRI slice. Figure 5b shows autofluorescence from channel 9 (pink) overlaid over background signal from channel 4 (green) for maximal contrast to visualize demyelination in the white matter; Fig. 5c shows Iba1 and PLP on the same section. Figure 5d shows another example, a lesion that developed 4–5 months before sacrifice (blue arrow, Fig. 5d) and another younger lesion that developed approximately a month later (dark yellow arrow, Fig. 5d) in the optic tract of EAE marmoset #9. The intensity of TSPO staining was found to be higher in the younger lesion (Fig. 5e), and the percentage of Iba1 + cells co-expressing TSPO was found to decline from 90.2% in the younger lesion to 40.4% in the older lesion. A similar pattern is also seen when comparing early lesions in animals 4–5 with chronic lesions in animals 6–9. In the lesions < 4 weeks old, over 88% of Iba1 + cells expressed TSPO, while in chronic lesions, the TSPO + fraction was < 70%. A comparison of fractional TSPO expression at various lesion ages in these animals is shown in Fig. 5f.

Using a linear mixed effects changepoint analysis to detect temporal patterns in TSPO expression in animals 4–9 and additive adjustment for the steroid treatment applied to animals 8–9, we found a statistically significant change in TSPO expression at 4–5 months of -12.8% (SEM = 3.0%, *p* = 0.01 with Bonferroni correction), indicating a decrease in TSPO levels in chronic lesions aged 4–5 months. Because two animals that were briefly treated with prednisolone in a concurrent study, an adjustment term was added to our general model to account for the potential effects of steroid treatment., but this was not found to be statistically significant (23.0%, SEM = 21.5%; *p* = 0.36).

### TSPO is low or absent in NAWM astrocytes in marmoset EAE, but elevated in chronic lesions with astrogliosis

**Fig. 6:**
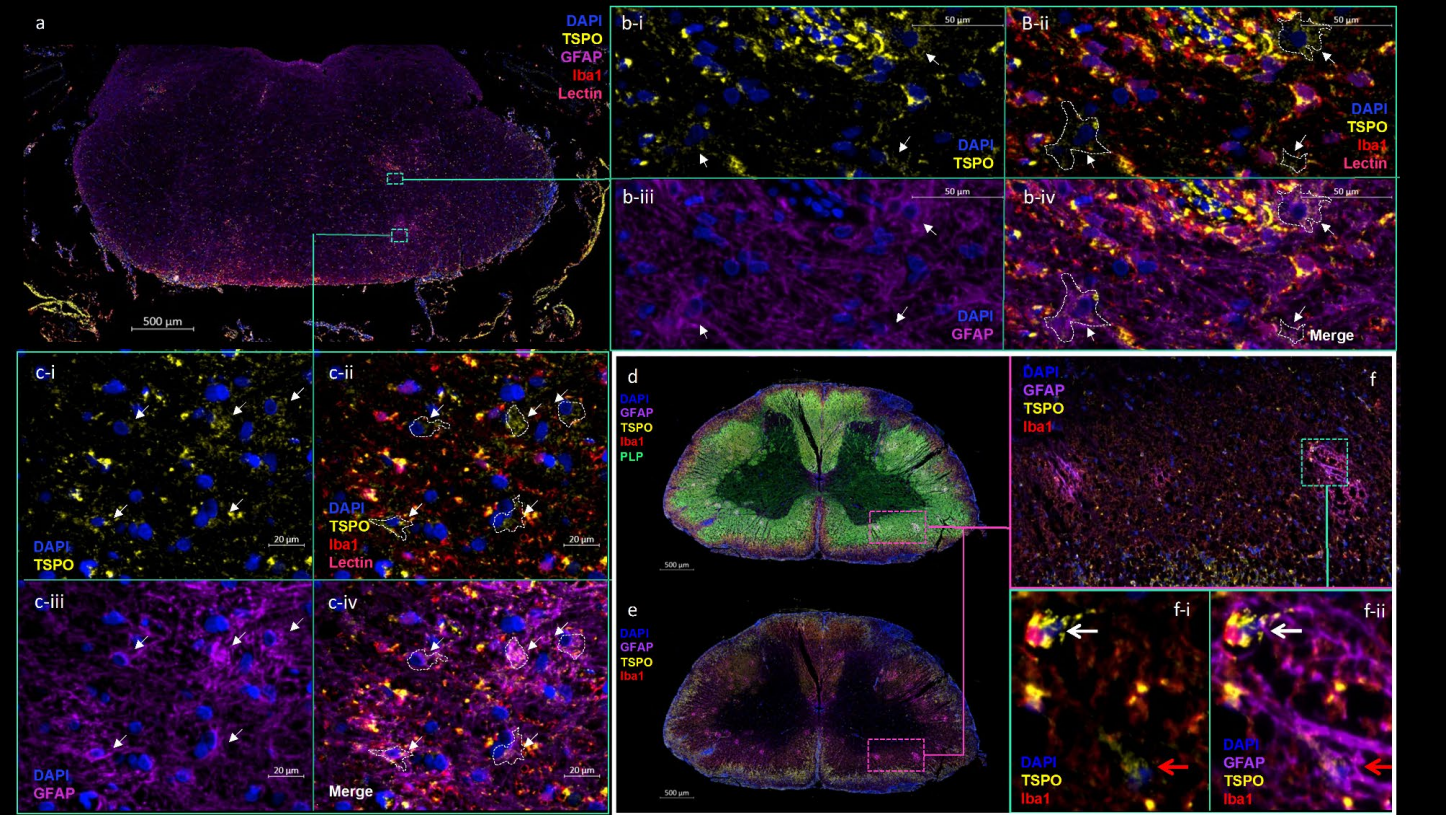
TSPO is found in GFAP + astrocytes in gliotic lesions of the spinal cord and brain in marmoset EAE. 6a) DAPI, TSPO, GFAP, Iba1, and lectin staining in the optic chiasm of Animal #3, with areas of interest indicated by green boxes. **6b-c)** A comparison from the corresponding area of interest from Animal #3 showing (i) DAPI and TSPO staining with arrows indicating areas of comparatively dim TSPO staining; (ii) DAPI, TSPO, Iba1, and lectin staining with arrows and white outlines indicating absence of Iba1 or lectin in previously identified areas of dim TSPO expression; (i) DAPI and GFAP staining with arrows indicating GFAP expression in previously identified areas of dim TSPO expression; and iv) merged DAPI, TSPO, Iba1, GFAP, and lectin staining with arrows and white outlines identifying cells that express GFAP and TSPO but not Iba1 or lectin. **6d)** DAPI, GFAP, TSPO, Iba1, and PLP staining from a section of the cervical spinal cord of animal #2 with multiple bright foci distributed throughout the white matter, with region of interest indicated by pink outline. **6e)** DAPI, GFAP, TSPO, and Iba1 staining for this section of cervical spinal cord, where the dominant color in these bright foci is found to be GFAP (in purple), with region of interest indicated by pink outline. **6f)** DAPI, GFAP, TSPO, and Iba1 staining in the indicated area of interest at higher resolution, where two dense clusters of GFAP staining are seen, indicating two distinct foci of astrogliosis, with lesion of interest indicated by green outline. **6f-i)** DAPI, TSPO, and Iba1 staining in the indicated lesion at higher resolution, where TSPO is observed both in an Iba1 + microglia/macrophage (white arrow), and a Iba1- cell (red arrow). **6f-ii)** DAPI, TSPO, PLP and Iba1 staining in the indicated lesion at higher resolution, where the previously indicated Iba1- cell is shown to be GFAP+ (red arrow)

Sections of CNS tissue from a healthy marmoset and multiple EAE marmosets were stained for TSPO, Iba1, lectin, DAPI, and GFAP to differentiate TSPO expression in microglia/macrophages from TSPO expression in astrocytes. GFAP + astrocytes in control animal #1 were found to be virtually devoid of TSPO (Fig. 1c). In lesions with significant astrogliosis, however, many GFAP + TSPO + cells are observed. Figure 6a shows DAPI, TSPO, Iba1, lectin, and GFAP staining in the optic chiasm of EAE animal #3, where multiple foci of high GFAP and Iba1 density are seen at sites of demyelination identified by PLP staining. Figure 6b-c shows DAPI, TSPO, Iba1, lectin, and GFAP staining in two of these foci. Relatively weakly TSPO + cells indicated by white arrows in 6b-i and 6c-i are shown to be devoid of Iba1 or lectin staining (Fig. 6b-ii and 6c-ii, white dotted lines) but do stain positively for GFAP (Fig. 6b-iii and 6c-iii, white arrows). These GFAP + cells are observed to exhibit a hypertrophic morphology typical of reactive astrocytes, i.e. larger, thicker cell bodies and processes, a higher number of processes, and an increased intensity of GFAP staining.

Astrocytic TSPO expression was also seen in EAE spinal cord tissue. Figure 6d shows DAPI, TSPO, PLP, Iba1, and GFAP staining from multiple foci in the cervical spinal cord of animal #2. Figure 6e shows DAPI, TSPO, and GFAP staining alone, revealing increased GFAP density in these foci. Figure 6f shows three of these foci of astrogliosis at higher resolution. On close examination of the rightmost lesion (green inset, Fig. 6f-i and f**-ii**), merged imaging of TSPO, Iba1, and DAPI demonstrates TSPO (yellow) in an Iba1+ (red) cell (white arrow, Fig. 6f-i), as well as an a Iba1- cell (red arrow, Fig. 6f-i). Merged imaging of TSPO, Iba1, DAPI, and GFAP reveals this Iba1-TSPO + cell to be a GFAP + astrocyte (red arrow, Fig. 6f-i i).

### Neuronal TSPO expression is increased in marmoset EAE

**Fig. 7:**
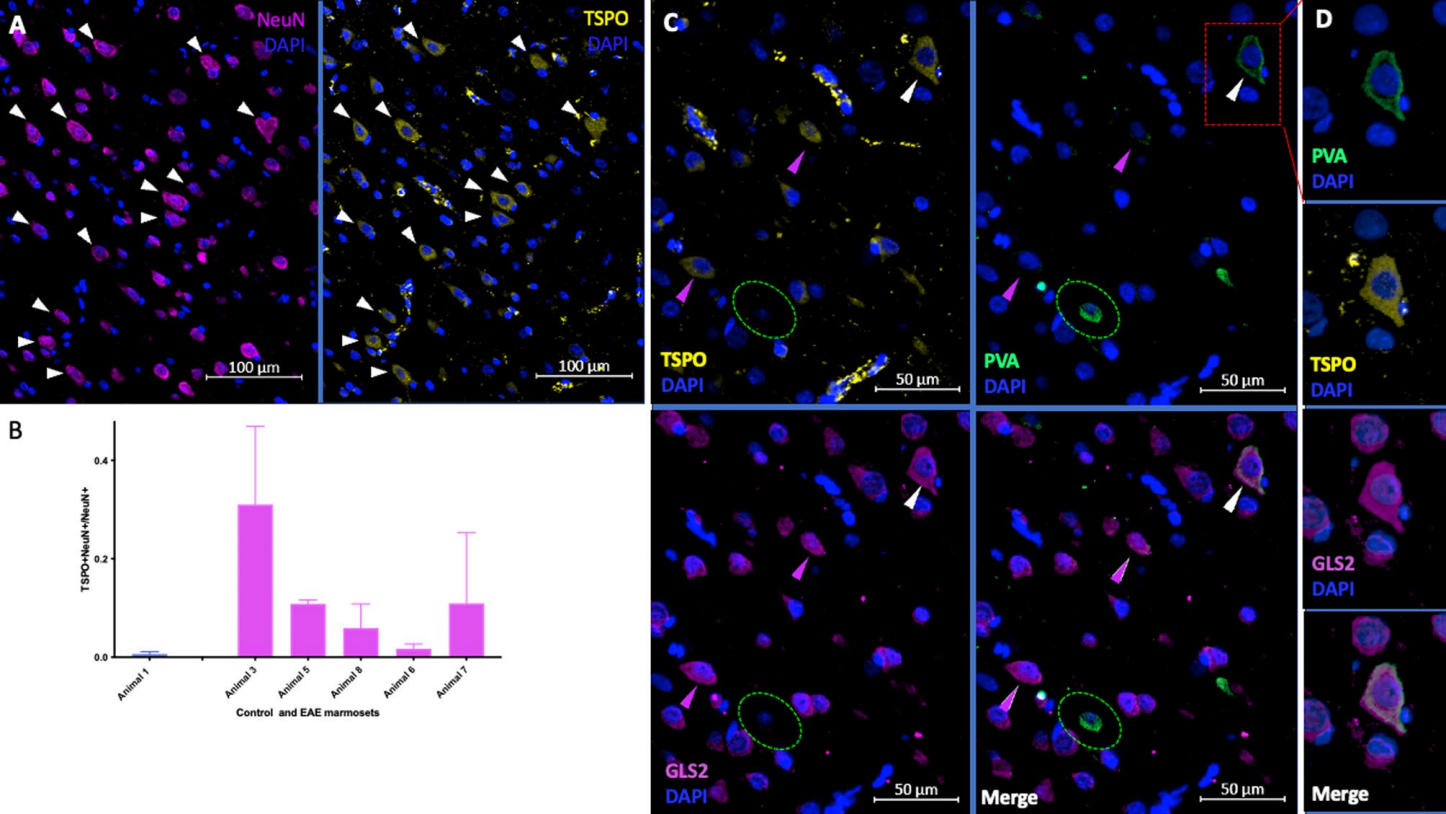
TSPO is expressed in a larger subset of neurons in marmoset EAE and is found in glutamatergic neurons but not interneurons. **7a)** Comparison of DAPI and TSPO staining with DAPI and NeuN staining in a region of cortical gray matter in EAE marmoset 3, which shows diffuse cytoplasmic TSPO expression in a subset of NeuN + cells (arrowheads). **7b)** Mean fraction of NeuN + cells co-expressing TSPO across all areas of interest in control marmoset #1 and EAE marmosets #4, #5, #6, #7 and #3. Error bars indicate SEM. **7c)** Comparison of TSPO staining with PVA and GLS2 staining from a section of brain tissue from EAE marmoset #3 that was stained with DAPI and TSPO and then stripped and re-stained for neuronal markers. Diffuse cytoplasmic TSPO staining is observed in GLS2 + PVA- glutamatergic neurons (pink arrowheads) and rare GLS2 + PVA + neurons (white arrowhead) but not PVA + GLS2- interneurons (green circle). **7d)** A representative example of a TSPO + GLS2 + PVA + neuron from Fig. 7c, shown at higher resolution

To investigate whether TSPO is expressed in neurons in healthy and inflamed CNS tissue, sections of brain tissue from control animal #1 and 5 EAE marmosets were stained for TSPO, Iba1, NeuN and DAPI. In the parenchyma of animal #1, TSPO expression was not observed in any NeuN + cells (Supplemental Fig. 1). In one region of the cortical gray matter of animal #3, however, TSPO was observed in the cell bodies of roughly one-third of NeuN + neurons (Fig. 7a). In contrast to the dense perinuclear vesicular pattern of TSPO staining observed in microglia/macrophages, TSPO staining in these neurons was of lower intensity and appeared to be distributed diffusely throughout the cytoplasm of the neuronal cell body (Fig. 7a, white arrowheads).

To quantify the percentage of neurons expressing TSPO, ROIs were selected in the right and left cortical gray matter, and the numbers of NeuN + cells co-expressing TSPO were counted manually. The fraction of NeuN + cells expressing TSPO in the right and left cortical gray matter of each animal are shown in Fig. 7b. In control marmoset #1, the percentage of neurons expressing TSPO was 0.6% (SEM = 0.3%); in the 5 EAE marmosets, the mean percentage was 12.1% (SEM = 5.0%). The difference between the means is 11.5% which is not statistically significant (*p* = 0.41, two-sided). The effect of laterality was tested against within-animal variation; both effects were non-significant.

To further probe the phenotype of TSPO + neurons in EAE, we had the opportunity to re-stain one tissue section from EAE animal #3. Briefly, an antibody stripping protocol was applied to this tissue section, which was then re-stained with antibodies against neuronal phenotypic markers GLS2, a glutaminase expressed in excitatory glutamatergic neurons, and parvalbumin (PVA), a calcium binding albumin protein expressed by inhibitory GABAergic interneurons. TSPO was found in 40.7% of GLS2 + PVA- neurons but was not observed in any PVA + GLS- cells. Examples of each neuronal type are shown in Fig. 7c. In addition, some cells were observed to have both bright GLS2 staining and dim PVA staining, an example of which is indicated by the white arrowhead in Fig. 7c and shown at higher resolution in Fig. 7d. TSPO was observed in these GLS2 + PVA + cells as well.

## Discussion

Understanding the cell types expressing TSPO in MS is important for our understanding of the pathological significance of TSPO-PET signal and its implications for disease status and therapeutic response. While rodent EAE is widely studied as a model of MS, rodents differ in many immunological and anatomical parameters, and recent studies have found notable differences in the mechanisms of TSPO expression in these species. Studies in rodents, for example, have found increased TSPO in activated microglia/macrophages, implying increased TSPO-PET signal to represent TSPO upregulation upon activation [15–17]. Human experiments, in contrast, have found no such increase, prompting the hypothesis that increased signal actually reflects increased myeloid cell density [18, 50]. Where rodent studies have found microglia to be principal contributors of TSPO in EAE [15, 51], human studies have found more prominent astrocytic TSPO expression in chronic disease [13]. Marmoset EAE is a potentially more clinically relevant model in which to probe the mechanisms of TSPO expression through the correlation of PET, MRI, and histopathology. To better understand the utility of this model as a proxy of human disease, we studied TSPO expression in different CNS cell types, examined its association with different markers of phagocytic activation, and measured phagocytic TSPO expression over time.

In control marmoset CNS tissue, we found that TSPO is expressed in the vascular endothelium, ependyma, and meninges, which is consistent with prior studies in rodent brain tissue [51]. TSPO expression was also examined in cells expressing Iba1, which is a calcium-binding protein expressed in microglia/macrophages[52]. Aside from a few Iba1 + cells observed in the lumina of cerebral blood vessels, the vast majority of these Iba1 + cells are located within the parenchyma. Notably, peripheral hemoglobin-haptoglobin receptor CD163 was confined to Iba1 + cells in the intravascular space, which is consistent with prior primate studies in macaques that have identified CD163 as a selective marker of perivascular macrophages that is not observed in parenchymal cells in healthy animals but is found intraparenchymally following BBB breakdown in simian-immunodeficiency virus (SIV)-infection [47, 48]. As there was no evidence of parenchymal CD163 expression to suggest BBB breakdown in control animal #1, the parenchymal Iba1 + cells in this animal were therefore presumed to be resident microglia. In contrast to prior rodent studies [51], which found no microglial TSPO expression in control rodent brain [51], we found TSPO in approximately two-thirds of Iba1 + cells, implying TSPO expression in homeostatic microglia, a finding that has also been observed in control human brain tissue [21].

In early marmoset EAE, TSPO becomes densely concentrated in EAE lesions, where it is intensely expressed by approximately 90% of microglia/macrophages, a pattern that is also reported in human disease [21]. These TSPO + microglia/macrophages include cells expressing CD74 or MRP14 (conventionally considered pro-inflammatory), cells expressing Arg1 or CD163 (conventionally considered pro-tolerogenic), and cells with mixed phenotypes expressing some combinations of the above markers. This is consistent with studies in MS and other forms of neurological injury that have shown TSPO co-localization with both pro- and anti-inflammatory markers in vivo [20]. However, we also observed a marked change in the expression of CD74, which was rarely expressed in control brain tissue but appeared in over 50% of Iba1 + microglia/macrophages in early lesions. This is consistent with previous reports demonstrating TSPO co-localization with HLA DR in human MS lesions [20, 21]. Similar increases were seen in the expression of MRP14 and CD163. The percentage of Iba1 + cells co-expressing Arg1 and TSPO, however, was not noticeably different.

These shifts could represent changes in the proportion of resident microglia relative to infiltrating peripheral macrophages. While hemoglobin-haptoglobin receptor CD163 was not expressed by microglia in control marmoset brain parenchyma, the receptor is expressed by Iba1 + cells in the context of BBB compromise in EAE. This may indicate either the induction of CD163 expression in resident microglia or the infiltration of CD163 + peripheral macrophages into the CNS. The increase in MRP14 may likewise reflect an increase in infiltrating peripheral immune cells, as MRP14 is also found in early active peripheral macrophages [45]. Further experiments are needed to distinguish resident microglia from infiltrating macrophages. A homeostatic microglial marker such as TMEM119 may be useful in this respect and has been successfully detected in fixed marmoset tissue [53]; however, it is also observed to downregulate upon microglial activation [54, 55]. CD206 would also be a useful marker for the distinction of perivascular macrophages from microglia [56], but currently we have not successfully validated a CD206 antibody for staining in fixed marmoset tissue.

The percentage of Iba1 + cells expressing TSPO in EAE lesions was observed to decrease with lesion age, as determined by serial MRI. While at least 88% of Iba1 + cells expressed TSPO in lesions < 4 weeks old, the percentage expressing TSPO was as low as 13% in lesions > 6 months old, consistent with studies in MS demonstrating a decline in the percentage of TSPO contributed by Iba1 + cells in chronic and inactive lesions [13, 21]. Using a linear mixed effects model to account for random and non-random sources of variation, lesion age at time of sacrifice was demonstrated to have a significant effect upon the fraction of Iba1 + cells expressing TSPO, with a statistically significant decrease occurring 4–5 months after lesion onset. This finding has significant implications for the clinical use of TSPO-PET imaging as a microglia/macrophage marker, suggesting TSPO becomes less sensitive as a marker of microglia/macrophages in chronic disease. A similar pattern was observed in one of the first PK11195 studies in MS, where TSPO signal was found to be high in lesions < 4 weeks old but low in chronic lesions [57, 58]; subsequent studies of chronic lesions have found variable tracer uptake [58]. One possible explanation is that TSPO is predominantly contributed by homeostatic microglia and infiltrating immune cells. Recent studies of MS lesion development suggest that chronic lesions contain fewer antigen-presenting cells, fewer T cells, and very few homeostatic microglia overall [45, 59, 60].

Additionally, human studies have found astrocytic TSPO expression to be more common in chronic diseases, including chronic MS [61], and we find that marmoset EAE recapitulates the astrocytic expression of TSPO in gliotic, chronic-appearing lesions. Specifically, TSPO expression is seen in GFAP + astrocytes with hypertrophic morphology in lesions where astrogliosis is extensive, but not in control tissue, NAWM, or lesions without astrogliosis. This feature distinguishes marmoset EAE from many rodent models of EAE, where astrocytic TSPO expression is inconsistently observed or absent [23, 62, 63]. Furthermore, the finding of decreasing microglial TSPO expression over time and higher astrocytic TSPO expression in lesions with signs of chronicity, i.e., astrogliosis, again is consistent with studies demonstrating decreases in the percentage of TSPO contributed by microglia/macrophages and increases in the percentage contributed by astrocytes in chronic active and inactive MS lesions [21].

Finally, we observed neuronal TSPO expression, a characteristic that has been observed in human neuroinflammatory disease but has not been confirmed in other animal models [64]. The percentage of neurons expressing TSPO was less than 1% in control CNS tissue, similar to mouse studies finding basal TSPO mRNA in 3% of neurons, but 12% on average in EAE animals. While further studies are needed to confirm the phenotype of TSPO + neurons in marmoset EAE, a multiplex study of neuronal phenotype markers in one EAE marmoset revealed TSPO expression in neurons expressing GLS2, a glutaminase found in excitatory glutamatergic neurons, but not in interneurons expressing parvalbumin (PVA). This appears to correlate with early studies on the cellular locations of neurosteroid synthesis, which found the necessary enzymatic machinery was expressed in glutamatergic pyramidal neurons but near absent in GABAergic interneurons [65]. Additionally, some TSPO + neurons were found to be express both GLS2 and PVA. The concurrent expression of both markers in these cells may indicate they are immature or regenerating neurons, consistent with in vitro studies demonstrating TSPO expression in neuronal precursor cells [64].

This study is the first to our knowledge to comprehensively characterize sources of TSPO expression in the CNS of the common marmoset, confirming its expression in the meninges, ependyma, vascular endothelium, and a subset of microglia in a control CNS tissue, as well as a larger percentage of microglia/macrophages and a subset of astrocytes and neurons in marmoset EAE. While microglia/macrophages were the principal contributors of TSPO density in acute lesions, we found that their contribution declines in chronic lesions and that astrocytic TSPO expression is more pronounced in lesions with astrogliosis. Finally, we characterized the immunophenotypes of TSPO-expressing microglia/macrophages in healthy and diseased primate brain. In keeping with studies in MS brain tissue [20], we found TSPO expression co-localizes with markers of antigen presentation and acute activation in marmoset EAE. However, we also find TSPO co-expression with markers of phagocytosis and immunosuppression, indicating TSPO expression is not restricted to a pro-inflammatory phenotype.

## Electronic supplementary material

Below is the link to the electronic supplementary material.

Supplementary Material 1

Supplementary Material 2

## Data Availability

No datasets were generated or analysed during the current study.

## Abbreviations

TSPO: 18-kDa Translocator Protein
EAE: Experimental Autoimmune Encephalomyelitis (EAE)
MS: Multiple Sclerosis
CNS: Central Nervous System
MOG: Myelin-Oligodendrocyte Glycoprotein
PLP: Proteolipid Protein
CFA: Complete Freund’s Adjuvant
NAWM: Normal-Appearing White Matter
NAGM: Normal-Appearing Gray Matter
