# Supplementary material for: Characteristics of TSPO expression in marmoset EAE
Source: J Neuroinflammation. 2025 Jan 27;22:19. doi: 10.1186/s12974-025-03343-4 (PMC11773908; doi:10.1186/s12974-025-03343-4)
Supplement: Supplementary file 1 — Supplementary Material 1 [file 12974_2025_3343_MOESM1_ESM.docx]

## Supplemental

**Table 1.** A complete list of validated primary antibodies used for the phenotyping of microglia and macrophages expressing TSPO in inflammatory lesions in the marmoset CNS.

| **Target** | **Clone** | **Host** | **Isotype** | **Company** | **Cat. No.** | **Dilution** |
| --- | --- | --- | --- | --- | --- | --- |
| PLP | Plpc1 | Mouse | IgG2a | Bio-Rad | MAC839G | 1:200 |
| TSPO | EPR5384 | Rabbit | IgG | Abcam | ab109497 | 1:100 |
| Iba1 | Polyclonal | Guinea Pig | IgG | Synaptic Systems | 234004 | 1:100 |
| Arginase 1 | ARG1 | Mouse | IgG3 | NSJ Biologicals | V2652 | 1:200 |
| CD74 | Polyclonal | Sheep | IgG | R&D Systems | AF3590 | 1:100 |
| MRP14 | MAC387 | Mouse | IgG1 | DAKO | M074701 | 1:100 |
| CD163 | 6E10.1G6 | Mouse | IgG2b | Novus Biologicals | NBP2-36494 | 1:100 |

**Table 2.**  A complete list of secondary antibodies used for the phenotyping of microglia and macrophages expressing TSPO in inflammatory lesions in the marmoset CNS.

| **Fluorophore** | **Secondary host** | **Host** | **Isotype** | **Company** | **Cat. No.** | **Dilution** |
| --- | --- | --- | --- | --- | --- | --- |
| DY395XL | Goat | Mouse | IgG2a | Li-Cor Biosciences | CSQ-0007-3 | 1:100 |
| Alexa Fluor 488 | Goat | Rabbit | IgG | ThermoFisher | A21131 | 1:200 |
| Alexa Fluor 546 | Goat | Guinea Pig | IgG | ThermoFisher | A11035 | 1:200 |
| Alexa Fluor 594 | Goat | Mouse | IgG3 | ThermoFisher | A21125 | 1:200 |
| Alexa Fluor 647 | Goat | Sheep | IgG | R&D Systems | A21450 | 1:200 |
| IRDye 680LT | Goat | Mouse | IgG1 | DAKO | 016-120-084 | 1:200 |
| IRDye 800CW | Goat | Mouse | IgG2b | Li-Cor Biosciences | 926-32352 | 1:100 |

**Table 3**. A complete list of validated primary antibodies used for the phenotyping of neurons and astrocytes expressing TSPO in inflammatory lesions in the marmoset CNS.

| **Target** | **Clone** | **Host** | **Isotype** | **Company** | **Cat. No.** | **Dilution** |
| --- | --- | --- | --- | --- | --- | --- |
| PCNA | PC8 | Mouse | IgG3 | GeneTex | GTX40237 | 1:200 |
| Collagen IV | Polyclonal | Rabbit | IgG | Abcam | ab6586 | 1:200 |
| NeuN | Polyclonal | Guinea Pig | IgG | Millipore | ABN90 | 1:500 |
| S100 | 15E2E2 | Mouse | IgG2a | Millipore | MAB079-1 | 1:200 |
| CNPase | 11-5B | Mouse | IgG1 | Millipore | MAB326 | 1:200 |
| NFH | Polyclonal | Chicken | IgY | Millipore | AB5539 | 1:500 |
| Lectin* | LEL | N/A | N/A | Vector Labs | B-1175 | 1:200 |
| MBP | 82-87 | Rat | IgG2a | Millipore | MAB386 | 1:200 |
| GFAP | Polyclonal | Mouse | IgG2b | BD Biosciences | 55630 | 1:200 |

**Table 4.**  A complete list of secondary antibodies used for the phenotyping of neurons and astrocytes expressing TSPO in inflammatory lesions in the marmoset CNS.

| **Fluorophore** | **Secondary host** | **Host** | **Isotype** | **Company** | **Cat. No.** | **Dilution** |
| --- | --- | --- | --- | --- | --- | --- |
| DyLight 405 | Goat | Mouse | IgG3 | Li-Cor Biosciences | CSQ-0007-3 | 1:100 |
| Alexa Fluor 430 | Goat | Rabbit | IgG | ThermoFisher | A11064 | 1:100 |
| Alexa Fluor 488 | Goat | Guinea Pig | IgG | ThermoFisher | A21151 | 1:100 |
| Alexa Fluor 546 | Goat | Mouse | IgG2a | ThermoFisher | A21133 | 1:200 |
| Alexa Fluor 594 | Goat | Mouse | IgG1 | ThermoFisher | A21125 | 1:200 |
| Alexa Fluor 647 | Goat | Chicken | IgY | ThermoFisher | Z21449 | 1:200 |
| StreptAvidin-PerCP | N/A | N/A | N/A | Jackson ImmunoResearch | 016-120-084 | 1:200 |
| IRDye 680LT | Goat | Rat | IgG2a | Li-Cor Biosciences | 926-68051 | 1:200 |
| IRDye 800CW | Goat | Mouse | IgG2b | Li-Cor Biosciences | 926-32219 | 1:100 |

***Table 5.****A summary of lesions included in this study, including identity of the animal in which the lesion was found, steroid treatment status, the beginning and end of the range in which the lesion was identified on MRI (in months from time of sacrifice) and the fraction of Iba1+ cells in this lesion expressing TSPO.*

| **Lesion #** | **Animal #** | **Treatment** | **MonthStart** | **MonthStop** | **TSPO.Ratio** |
| --- | --- | --- | --- | --- | --- |
| 1 | 6 | None | -8 | -7 | 0.1699695 |
| 2 | 6 | None | -7 | -6 | 0.13341 |
| 3 | 6 | None | -6 | -5 | 0.1589041 |
| 4 | 6 | None | -3 | -2 | 0.5070423 |
| 5 | 6 | None | -3 | -2 | 0.2063712 |
| 6 | 6 | None | -6 | -5 | 0.1875 |
| 7 | 6 | None | -6 | -5 | 0.1942801 |
| 8 | 6 | None | -8 | -7 | 0.2486911 |
| 9 | 6 | None | -4 | -3 | 0.2350209 |
| 10 | 6 | None | -4 | -3 | 0.2582448 |
| 11 | 6 | None | -3 | -2 | 0.3400277 |
| 12 | 6 | None | -7 | -6 | 0.19444 |
| 13 | 7 | None | -8 | -6 | 0.660571 |
| 14 | 7 | None | -8 | -6 | 0.643208 |
| 15 | 4 | None | -1 | 0 | 0.9666766 |
| 16 | 4 | None | -1 | 0 | 0.9540216 |
| 17 | 5 | None | -1 | 0 | 0.8828039 |
| 18 | 8 | Steroid | -1 | 0 | 0.961887 |
| 19 | 8 | Steroid | -3 | -1 | 0.791196 |
| 20 | 8 | Steroid | -3 | -1 | 0.922923 |
| 21 | 9 | Steroid | -4 | -3 | 0.902 |
| 22 | 9 | Steroid | -5 | -4 | 0.4044 |

**Table 6.**  A summary of the patterns of marker expression observed in Animal #1, the number of cells expressing the given combination, the percentage of those cells expressing TSPO.

| Marker expression pattern | No. of cells | % TSPO+ |
| --- | --- | --- |
| Arg1+ CD163+ CD74+ MRP14+ | 54 | 100.00 |
| Arg1+ CD163- CD74- MRP14+ | 4 | 0.00 |
| Arg1+ CD163+ CD74- MRP14- | 22 | 100.00 |
| Arg1+ CD163+ CD74+ MRP14- | 215 | 86.98 |
| Arg1+ CD163- CD74+ MRP14- | 2830 | 88.87 |
| Arg1+ CD163- CD74- MRP14- | 3294 | 85.37 |
| Arg1+ CD163- CD74+ MRP14+ | 76 | 100.00 |
| Arg1- CD163- CD74+ MRP14- | 148 | 87.84 |
| Arg1- CD163- CD74+ MRP14+ | 22 | 100.00 |
| Arg1- CD163- CD74- MRP14+ | 14 | 100.00 |
| Arg1- CD163+ CD74+ MRP14+ | 52 | 100.00 |
| Arg1- CD163+ CD74- MRP14+ | 2 | 100.00 |
| Arg1- CD163+ CD74- MRP14- | 382 | 68.06 |
| Arg1- CD163- CD74- MRP14- | 27230 | 69.29 |

.

**Table 7.**  A summary of the patterns of marker expression observed in an acute lesion from Animal #4, the number of cells expressing the given combination, the percentage of those cells expressing TSPO.

| Marker expression pattern | No. of cells | % TSPO+ |
| --- | --- | --- |
| Arg1+ CD163+ CD74+ MRP14+ | 54 | 100.00 |
| Arg1+ CD163- CD74- MRP14+ | 4 | 0.00 |
| Arg1+ CD163+ CD74- MRP14- | 22 | 100.00 |
| Arg1+ CD163+ CD74+ MRP14- | 215 | 86.98 |
| Arg1+ CD163- CD74+ MRP14- | 2830 | 88.87 |
| Arg1+ CD163- CD74- MRP14- | 3294 | 85.37 |
| Arg1+ CD163- CD74+ MRP14+ | 76 | 100.00 |
| Arg1- CD163- CD74+ MRP14- | 148 | 87.84 |
| Arg1- CD163- CD74+ MRP14+ | 22 | 100.00 |
| Arg1- CD163- CD74- MRP14+ | 14 | 100.00 |
| Arg1- CD163+ CD74+ MRP14+ | 52 | 100.00 |
| Arg1- CD163+ CD74- MRP14+ | 2 | 100.00 |
| Arg1- CD163+ CD74- MRP14- | 382 | 68.06 |
| Arg1- CD163- CD74- MRP14- | 27230 | 69.29 |

**Table 8.**  A summary of the patterns of marker expression observed in an acute lesion from Animal #5, the number of cells expressing the given combination, the percentage of those cells expressing TSPO. ***Michelle

| Marker expression pattern | No. of cells | % TSPO+ |
| --- | --- | --- |
| Arg1+ CD163+ CD74+ MRP14+ | 192 | 100.00 |
| Arg1+ CD163- CD74- MRP14+ | 35 | 0.00 |
| Arg1+ CD163+ CD74- MRP14- | 136 | 100.00 |
| Arg1+ CD163+ CD74+ MRP14- | 183 | 86.98 |
| Arg1+ CD163- CD74+ MRP14- | 17 | 88.87 |
| Arg1+ CD163- CD74- MRP14- | 3 | 85.37 |
| Arg1+ CD163- CD74+ MRP14+ | 18 | 100.00 |
| Arg1- CD163- CD74+ MRP14- | 10 | 87.84 |
| Arg1- CD163- CD74+ MRP14+ | 15 | 100.00 |
| Arg1- CD163- CD74- MRP14+ | 5 | 100.00 |
| Arg1- CD163+ CD74+ MRP14+ | 143 | 100.00 |
| Arg1- CD163+ CD74- MRP14+ | 109 | 100.00 |
| Arg1- CD163+ CD74- MRP14- | 2 | 68.06 |
| Arg1- CD163- CD74- MRP14- | 23 | 69.29 |
