# Supplementary figures and images for: Characteristics of TSPO expression in marmoset EAE
Source: J Neuroinflammation. 2025 Jan 27;22:19. doi: 10.1186/s12974-025-03343-4 (PMC11773908; doi:10.1186/s12974-025-03343-4)

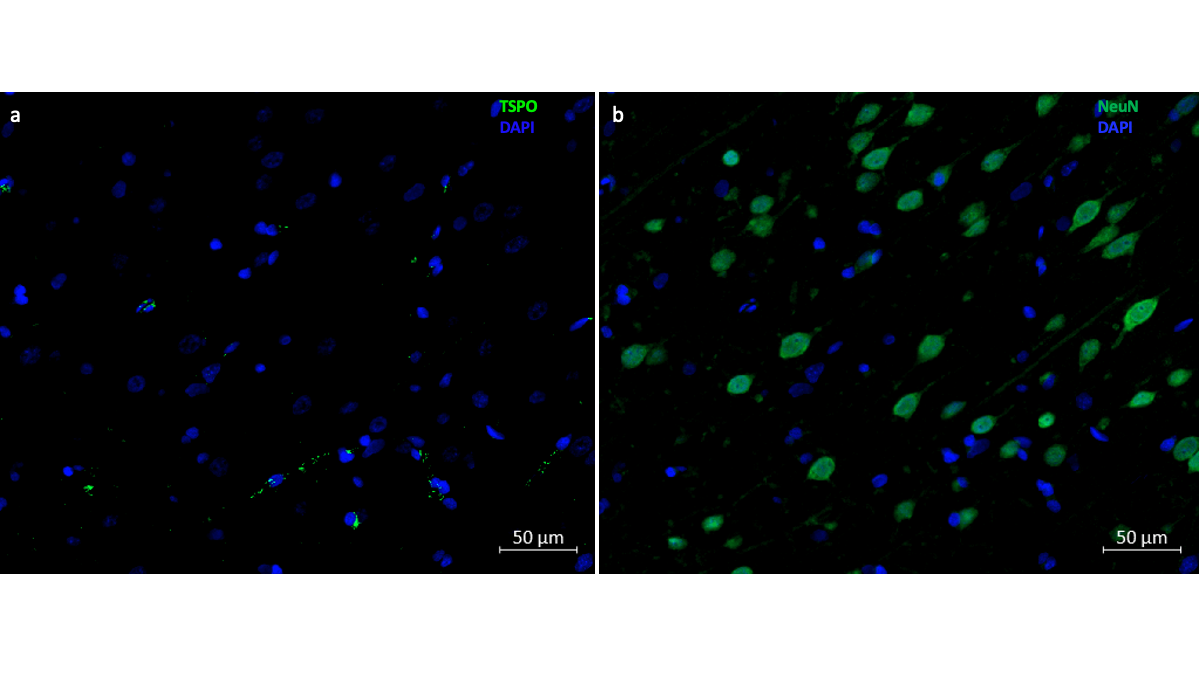

Supplement: Supplementary file 2 — Supplementary Material 2 [file 12974_2025_3343_MOESM2_ESM.tiff]
